# Integrative oncology along with systemic cancer therapies in advanced bladder and prostate cancer: metabolic optimization guiding the shift from palliative care to clinical remission – a case report

**DOI:** 10.3389/fonc.2026.1755406

**Published:** 2026-05-18

**Authors:** Pradeep M. K. Nair, Ayyappan Palanisamy, Sivaranjani Sekar, Shanmugam Sudarshan, Elangovan Karthika, Muniappan Devibala, Maruthanayagam Saranya, Thangavelu Ramasamy, Manickam Mahalingam

**Affiliations:** Department of Integrative Oncology, Mirakle Integrated Health Centre, Pollachi, India

**Keywords:** cancer metabolism, hyperthermia, nutrition therapy, patient-reported outcome measures, tumor microenvironment, urinary bladder neoplasms

## Abstract

Bladder cancer, particularly in older adults, poses significant therapeutic challenges due to its high recurrence rates, metastatic potential, and the toxicity associated with standard treatments. This case report describes a 75-year-old male with advanced muscle-invasive urothelial carcinoma of the bladder, exhibiting metastases to the prostate and lymph nodes. Following intolerance to conventional palliative chemotherapy, the patient underwent a structured integrative oncology protocol (systemic cancer therapies combined with adjuvant complementary therapies) that combined metabolic therapies, oncothermia, high-dose intravenous vitamin C, targeted nutraceutical supplementation, acupuncture, laser therapy, diet therapy, and yoga-based interventions. After initial metabolic optimization, an oral chemotherapy regimen comprising Afatinib, Axitinib, Relugolix, and Abiraterone was initiated. The patient demonstrated excellent tolerance to treatment, significant symptomatic relief, normalization of cancer-related biomarkers, and marked metabolic response with stable residual metabolic activity on PET-CT within six months. Quality of life measures improved substantially, and prognostic indices, including the Chuang Survival Score, reflected enhanced survival potential. This report underscores the potential of systemic cancer therapies along with integrative oncology to improve clinical outcomes, mitigate treatment-related toxicity, and enhance quality of life in advanced bladder cancer. While the findings are encouraging, they warrant validation through larger, controlled studies to better define the role of integrative approaches within the contemporary oncology framework.

## Introduction

Carcinoma of the urinary bladder is one of the most common urological malignancies affecting older adults and accounts for approximately 3.9% of all cancers in men (1). Transurethral resection of a bladder tumor (TURBT) is a widely used procedure for both the diagnosis and treatment of bladder cancer. Besides this, chemotherapy, cystectomy, and radiation are also used as per the staging and severity of the bladder cancer (2). The survival rate among bladder cancer patients varies by country and ranges between 60% and 70% following active treatment (3). However, despite the effectiveness of present conventional therapies, reports indicate a high rate of recurrence, metastasis and significant costs associated with the treatments of bladder cancer (3, 4). Further, bladder cancer is regarded as 14^th^ leading cause of cancer-related deaths (5).

Most management strategies for bladder cancer primarily focus on correcting genetic dysfunctions. Emerging evidence highlights the importance of addressing underlying metabolic dysfunctions, which may precede or interact with genetic alterations and contribute to cancer development and progression, including findings previously reported by our group (6, 7). Addressing cancer comprehensively by integrating both genetic and metabolic aspects is considered a more holistic approach, as it not only provides symptom relief but also enhances the quality of life for patients (6, 8). However, there is insufficient data to support this hypothesis, except for a few reports (9).

The use of complementary medicine approaches in bladder cancer remission and survival alongside with conventional care is limited, with only a few studies reporting the benefits of intravenous nutrition, calorie restriction, dietary modifications, and nutraceuticals. Furthermore, given the high cost of bladder cancer treatment, it is essential to adopt a holistic approach that addresses the root cause and helps prevent recurrence. The present report discusses a case of a 75-year-old male, diagnosed with carcinoma of urinary bladder with metastasis on the prostate and lymph nodes, treated with an integrative medicine protocol along with systemic cancer therapies focused on cancer metabolism.

### Case presentation

A 75-year-old male presented with complaints of hematuria, abdominal distension, and pain in the sacroiliac region. He also reported severe fatigue, heartburn, and loss of appetite. He had no known comorbidities such as diabetes, hypertension, or other metabolic disorders. In October 2023, the patient was diagnosed with high-grade muscle-invasive urothelial carcinoma based on histopathological examination following TURBT at a conventional oncology hospital. The findings were consistent with a bladder primary, with prostatic involvement interpreted as secondary infiltration rather than a distinct primary prostate malignancy. A positron emission tomography (PET-CT) scan (November 2023) further revealed hypermetabolic lesions in the right vesicoureteric junction and the left lobe of the prostate gland, along with multiple pelvic and retroperitoneal metastases.

His treating oncologist, considering the advanced nature of the disease, disseminated malignancy and poor prognosis of his condition recommended 4 cycles of palliative chemotherapy regimen with gemcitabine and carboplatin. He developed severe side effects to the palliative chemotherapy (after the first cycle) like muscle weakness, electrolyte imbalance, pain in the abdomen, nausea and lack of appetite that lasted for 2 weeks.

The patient then decided against undergoing chemotherapy and went for self-herbal remedies for 4 months (October 2023- March 2024). He developed severe pain in his hip which limited his movement and also had complaint of dark black discolouration of urine. He visited our integrative oncology center in March 2024 with similar complaints and was admitted as an inpatient.

### Clinical findings

His weight was 72 kg, height 165 cm, and BMI 26.4 kg/m². He was not on any medications at the time of admission and had stable vital signs.

### Diagnostic focus and assessments

On admission (baseline), a complete blood count, liver function tests, kidney function tests, tumor markers, D-dimer, ferritin, lactate dehydrogenase, blood sugar profile, vitamin D, vitamin B12, parathyroid hormone, and ionized calcium levels were measured. These tests were repeated at intervals ranging from 1 to 3 months. A PET-CT scan was performed periodically to assess the patient’s prognosis. **Table 1** presents the values of all parameters measured across different time points.

**Table 1:** Changes in the biochemical and cancer markers across time points.

Time points	RBC	HB	PCV	TC	N	L	E	FER	CEA	PSA	LDH	D-dimer	CRP	Weight	BMI	Vit-D	SGOT	SGPT	ALP	Urea	Creatinine
Mar 2024	4.24	13.8	40.3	8310	51	43.7	1.4	266.4	4.57	10.08	326	221	2.3	69	25.3	24.9	27.46	12.63	105.1	14.34	0.86
May 2024	3.89	13.4	36.7	6570	42.40	51.50	1.20	202.4	4.65	12.97	201	222	1.8	68	25	31	16.50	9.02	94.9	15.47	0.73
Jul 2024	3.96	13.9	40	6090	40.30	52.80	1.40	185.5	3.96	16.31	192	388	1.9	67	24.6	63.6	16.72	8.93	115.7	15.44	0.74
Aug 2024	4.30	14.7	42.5	6890	42.80	51.4	1.40	220.7	4.13	19.30	207	314	2.0	66	24.2	77.5	21.05	10.12	107	14.67	0.89
Sep 2024	3.75	13.1	37.6	7220	43.20	49.80	0.30	352.5	4.83	0.349	206	220	4	66	24.2	NE	18.54	10.25	96.5	22.98	0.76
Feb 2025	4.00	13.4	39.4	8090	53.9	40	1.20	236.0	2.73	0.048	224	246	2	66	24.2	38.7	24.07	8.54	144.8	19.20	0.67

RBC- Red blood cells (million/μL); Hb-Hemoglobin (g/dL); PCV- Packed cell Volume (%); TC-Total leucocyte count (Cells/μL); N- Neutrophils (%); L-lymphocytes (%); E- Eosinophils (%); FE- Ferritin (ng/mL); CEA- Carcinoembryonic antigen (ng/mL); PSA-Prostate specific Antigen (ng/mL); LDH- lactate Dehydrogenase (U/L); D-Dimer (ng/mL); CRP- C-reactive protein (mg/dL); BMI- Body Mass Index (Kg/m3); Vit D- Vitamin D (ng/mL); SGOT -Serum Glutamate Oxaloacetate Transaminase (U/L); SGPT -Serum Glutamate Pyruvate Transaminase (U/L); ALP -Alkaline Phosphatase (U/L); Urea (mg/dL); Creatinine (mg/dL).

The quality of life of the patient was measured using European Organization for the Research and Treatment of Cancer Quality of Life Questionnaire (EORTC QLQ-C30), a 30-item instrument designed to measure quality of life in all cancer patients (10). Edmonton symptom assessment scale (11), a 9-item scale that quantifies the predominance of common cancer-related symptoms was used to assess the changes in symptoms of our patient. The patient’s survival rate was assessed using the Chuang survival score scale, a prognostic tool that evaluates survival chances based on eight parameters, where a higher score indicates a poorer prognosis (12). **Table 2** illustrates the changes in QoL, symptoms and survival score. **Table 3** presents the PET-CT outcomes assessed using European Organization for Research and Treatment of Cancer (EORTC) criteria for metabolic response, used in oncology, a standardized method for evaluating fluorine-18 fluorodeoxyglucose (FDG) PET responses in oncology (13). These parameters were evaluated to assess both the functional, genetic and metabolic markers of cancer.

**Table 2:** Changes in quality of life, symptoms and survival score across time points.

Time points	Edmonton symptom assessment Scale									EORTC QLQ-C30 scale			Chuang survival score
	Nausea	Depression	Anxiety	Drowsy	Appetite	Well-being	Breathlessness	Pain	Tiredness	Global score	Functional score	Symptom score	
March 2024	0	4	4	0	1	1	0	3	0	58.3	68.8	25.6	1
Jul 2024	1	4	8	2	3	4	1	4	4	83.3	83	20.3	1
Aug 2024	0	0	0	2	0	0	0	1	0	83.3	77.7	15.3	0.2
Sep 2024	0	0	0	0	0	3	0	0	0	66.6	97.7	10.2	0
Feb 2025	0	0	0	0	0	0	0	0	0	83.3	71.1	17.9	0

European Organization for the Research and Treatment of Cancer Quality of Life Questionnaire -EORTC QLQ-C30.

**Table 3:** Comparison of metabolic response in the PET scans across time points as per European Organization Research and Treatment of cancer criteria.

Lesion site	Nov 2023 (SUV max)	Jan 2024 (SUV max)	% Change	June 2024 (SUV max)	% Change	Nov 2024 (SUV max)	% Change	April 2025 (SUV max)	% Change	Metabolic response
Urethra	8.71	NFDGU	-100% ☑	NFDGU	-100% ☑	NFDGU	-100% ☑	NFDGU	-100% ☑	CMR
Urinary bladder	12.47	NFDGU	-100% ☑	5.7	-54.29% ☑	NFDGU	-100% ☑	NFDGU	-100% ☑	CMR
Bladder neck	8.54	8.54	0%	NFDGU	-100% ☑	NFDGU	-100% ☑	NFDGU	-100% ☑	CMR
Prostate	5.78	3.12	-46.02% ☑	4.4	-23.88%	NFDGU	-100% ☑	NFDGU	-100% ☑	CMR
REI	2.69	5.29	+96.65% ▵	4.8	+78.4% ▵	4.8	+78.4% ▵	2.4	-10.4% ▿	SMD
LEI	2.63	4.39	+66.92% ▵	4.4	+67.3% ▵	4.4	+67.3% ▵	2.6	-1.1% ▿	SMD
Common iliac	2.0	NFDGU	-100% ☑	4.6	+130% ▵	4.6	+130% ▵	NFDGU	-100% ☑	CMR
Retro-peritoneum	0.92	NFDGU	-100% ☑	5.6	+508.7% ▵	2.6	+182.6% ▵	NFDGU	-100% ☑	CMR

NFDGU, No FDG Uptake; REI, Right External Illiac; LEI, Left External Illiac; CMR, Complete Metabolic Response; SMD, Stable Metabolic Disease; PMR, Partial Metabolic Response; PMD, Progressive Metabolic Disease; CMR, Complete Metabolic Response (No FDG uptake); SMD: <25% increase or decrease.

## Methods

After obtaining written informed consent and conducting a detailed consultation, an integrative oncology protocol was planned by a team of integrative physicians, including experts in yoga and naturopathy, homeopathy, Siddha, general medicine, and medical oncology. The protocol consisted of 43 days of inpatient treatment across three time points (24 days, 12 days, and 7 days) from March 2024 to March 2025, along with biweekly outpatient visits during the inpatient treatment window. The timeline of events is presented in supplementary file.

### Therapeutic focus and assessment

This patient was dealt using a multimodular therapeutic approach combining both systemic cancer therapies and integrative medicine therapies. The primary therapeutic focus of the administered integrative oncology protocol was to address underlying mitochondrial dysfunction, oxidative stress, tissue hypoxia, impaired autophagy, and an acidic tumor microenvironment, which are considered key contributors to cancer development and progression (6). In addition to these metabolic priorities, this treatment protocol also intended to address the subsequent genetic aberrations in cancer. The treatment protocol included oral chemotherapy (Afatinib, Axitinib, Relugolix and Abiraterone), oncothermia, nutraceutical supplements, intravenous nutrition, high dose vitamin C, laser therapies, acupuncture, yoga, diet therapy, mud therapy and hydrotherapy. A detailed, structured treatment protocol including dosage, frequency, duration, and rationale for each intervention has been provided in Supplementary Table to enhance reproducibility.

### Follow-up

Apart from the inpatient and outpatient visits, the patient was advised to follow a strict millet-based diet, eliminating sugar, artificial sweeteners, maida, refined oils, milk, and rice. Blood parameters were measured at two-month intervals, with the last follow-up assessment conducted at six months, while PET-CT scans were repeated every six months.

### Outcomes

The patient responded favorably to the integrative oncology protocol (combination of systemic cancer therapies and complementary therapies), demonstrating a progressive reduction in symptoms, a notable improvement in quality of life, and consistent normalization of cancer-related blood markers over the course of treatment. The Chuang survival score improved from 1 to 0, indicating a positive shift in prognosis and enhanced survival potential. Serial PET-CT scans revealed a gradual and sustained reduction in hypermetabolic lesions, reflecting a continued therapeutic response. Notably, the most recent PET-CT scan conducted in November 2024 and April 2025 showed a complete metabolic response in the primary lesion and minimal metabolic activity in the iliac lymph nodes (i.e., marked metabolic response with stable residual metabolic activity), indicative of clinical remission.

**Figure 1:**
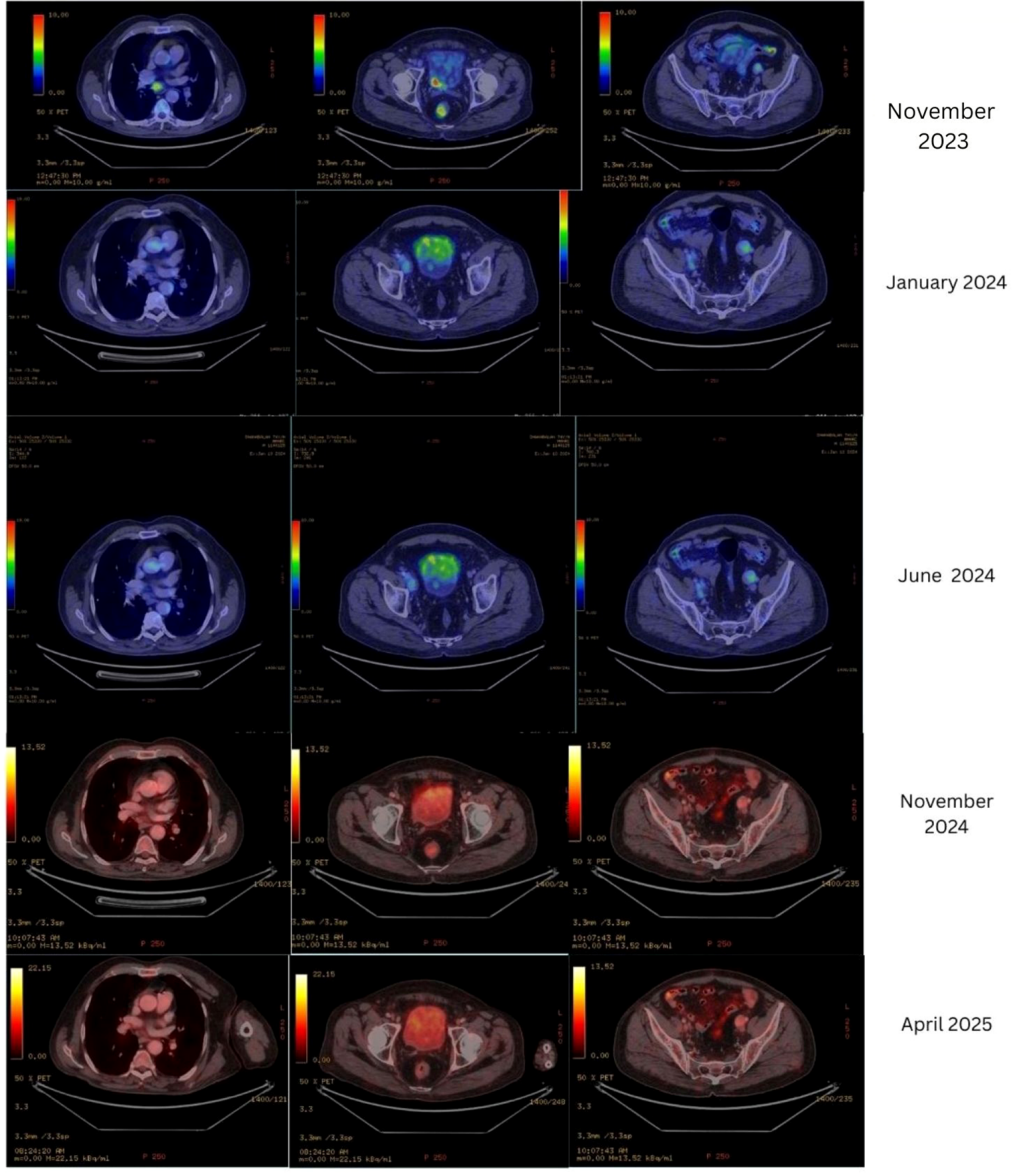
Changes in PET-CT across time points.

The detailed biochemical, quality of life and changes in PET scans are presented in **Table 1**-**3** respectively. **Figure 1** illustrates the serial PET-CT scans across different time points, highlighting the regression of hypermetabolic activity.

## Discussion

This case report describes a patient with advanced muscle-invasive urothelial carcinoma of the bladder with prostatic and nodal involvement who demonstrated a marked metabolic response following a comprehensive multimodal treatment approach. While the clinical course observed is encouraging, it is important to interpret these findings with caution. Given the concurrent use of multiple systemic anticancer agents alongside integrative interventions, it is not possible to attribute the observed outcomes to any single modality. Rather, the case highlights the potential feasibility of a multimodal strategy integrating conventional and supportive therapies in a patient who was intolerant to initial chemotherapy.

Bladder cancer remains a significant oncological challenge globally, particularly in older adults (1). Although conventional therapies such as chemotherapy, radiation, and surgery form the cornerstone of treatment, recurrence and metastasis remain common. In this case, the standard chemotherapy regimen (gemcitabine and carboplatin) was poorly tolerated by the patient, prompting a shift toward integrative approaches. The patient’s decision to discontinue chemotherapy due to adverse effects is consistent with existing literature that highlights the high burden of toxicity and diminished quality of life associated with palliative chemotherapy in elderly patients with advanced cancer (14).

The integrative oncology protocol employed in this case was unique in its multimodal nature, combining conventional, traditional, and complementary therapies. The therapeutic approach was guided by a holistic understanding of cancer biology, targeting not only genetic mutations but also metabolic dysfunctions such as mitochondrial dysregulation, oxidative stress, hypoxia, impaired autophagy, and the acidic tumor microenvironment. Emerging evidence suggests that these metabolic abnormalities are not merely consequences but potential drivers of cancer progression (6, 7). This paradigm shift in understanding cancer etiology reinforces the rationale for metabolic interventions in oncology.

Oncothermia, one of the core modalities used, is a loco-regional hyperthermia technique that selectively targets malignant cells while sparing healthy tissue. Oncothermia has shown to enhance cellular immunity, disrupt tumor metabolism, and improve drug delivery (15, 16). The patient also received high-dose vitamin C, a therapy gaining renewed interest for its pro-oxidant effect at pharmacologic concentrations, which selectively induces cytotoxicity in cancer cells while promoting normal cell survival (17).

In this case report of advanced bladder and prostate cancer treated with an integrative oncology approach, the adjunctive interventions, including acupuncture, laser/magnet therapy, nutraceuticals, diet therapy, and yoga were applied with the goals of reducing symptom burden, enhancing immune function, modulating metabolism, and improving psychological well-being. While conventional oncologic treatments address tumor burden directly (e.g., via cytotoxic or targeted therapy), these complementary interventions may influence the broader biological “terrain” including metabolic homeostasis, inflammation, immune surveillance, and stress-related pathways which may in turn modulate tumor behavior, treatment tolerance, and overall patient resilience (18). Previous studies have also highlighted the benefits of such multimodal integrative approaches in improving quality of life and treatment tolerance among cancer patients (19–21).

Notably, the millet-based, low-glycemic, and anti-inflammatory diet prescribed to the patient may have contributed to stabilizing the tumor microenvironment and improving metabolic parameters. Nutritional oncology has long advocated for dietary modulation to suppress systemic inflammation, reduce insulin resistance, and optimize nutrient delivery, all factors implicated in tumorigenesis (22). Recent advances in cancer biology have re-emphasized that tumor growth and survival are deeply dependent on metabolic supply, nutrients, oxygen, and the metabolic state of the host. Nutrient availability and metabolic signaling in the tumor microenvironment affect proliferation, survival, and therapy response (23).

Moreover, combining functional/nutrient-rich diets with lifestyle interventions (e.g., exercise, yoga) seems promising for modulating mitochondrial function, redox homeostasis, inflammation, and immune signaling, mechanisms that may be relevant not just for general health but potentially for controlling cancer progression (24). Thus, in our case, tailored diet therapy and nutraceutical support might have contributed to creating a metabolic milieu less favorable to aggressive tumor growth, supporting systemic health, and improving treatment tolerance.

Immune suppression and chronic inflammation are recognized contributors to tumor progression and poor outcomes. The metabolic–immune–inflammatory axis is increasingly viewed as a critical determinant of cancer behavior (6, 7). Evidence suggests that mind-body interventions such as yoga, stress-reduction, and other integrative therapies can attenuate stress responses, which otherwise may impair immune surveillance (e.g., reducing the activity of cytotoxic immune cells) (25). Furthermore, immunomodulatory effects of Acupuncture in cancer patients are also well documented. A recent meta-analysis found significant increases in markers such as interferon-γ, natural killer (NK) cells, CD3/CD4 counts, and favorable changes in inflammatory cytokines (e.g., reduced IL-6, IL-1, CRP) after acupuncture interventions (26). By improving immune parameters, acupuncture, together with other supportive therapies, might help restore immune surveillance and anti-tumor immune capacity, potentially assisting in control of residual disease or micrometastases. Nevertheless, these need to be validated by future studies.

Lifestyle interventions combining functional foods and physical activity (or movement-based therapies) have been shown to modulate redox homeostasis, mitochondrial integrity, inflammation, and neuroimmune signaling, potentially influencing both systemic health and cancer-specific pathways (24). In our case, the use of yoga, diet therapy, and nutraceuticals may have had a synergistic effect to reduce metabolic stress, improve mitochondrial and cellular energetics, lessen oxidative stress, and thereby create a host environment less conducive to tumor proliferation and progression.

After initial metabolic therapies, we incorporated a regimen of four oral chemotherapeutic agents—Afatinib, Axitinib, Relugolix, and Abiraterone—each targeting distinct oncogenic pathways associated with urinary bladder and prostate cancer. These agents are primarily known for their roles in inhibiting receptor tyrosine kinases, suppressing androgen-driven tumor growth, and modulating angiogenesis, thereby contributing to a multimodal therapeutic approach. The patient tolerated the oral chemotherapy regimen exceptionally well, without experiencing significant adverse effects typically associated with such treatments. This favorable outcome can be attributed to comprehensive nutritional optimization prior to the initiation of chemotherapy. As part of the integrative protocol, the patient underwent metabolic correction through targeted intravenous nutrition, nutraceutical supplementation, mitochondrial support therapies, and a personalized anti-inflammatory diet. These interventions likely enhanced his physiological resilience, improved immune competence, stabilized electrolyte balance, and optimized organ function, thereby mitigating the toxicity commonly observed with chemotherapeutic agents.

The patient demonstrated sustained improvement across multiple objective and subjective measures: biochemical markers of cancer normalized over time, quality of life improved (as indicated by EORTC QLQ-C30 and Edmonton Symptom Assessment Scale scores), and PET-CT findings showed marked metabolic response with stable residual metabolic activity by the sixth month of follow-up. Importantly, the Chuang Survival Score improved from 1 to 0, highlighting a reversal of poor prognostic indicators. These findings underscore the potential of integrative oncology to serve as an adjunctive strategy for patients who demonstrate intolerance to conventional therapies, thereby facilitating improved clinical outcomes and quality of life.

Despite these promising results, several limitations must be acknowledged. First, as a single-case report, these findings cannot be generalized without caution. Second, the lack of randomization, control, or blinding precludes definitive conclusions about causality. Third, the patient’s use of herbal remedies prior to integrative treatment introduces potential confounding variables. Also, many integrative therapies such as magnet therapy or certain energy/laser-based modalities may lack robust clinical trials with hard endpoints (e.g., survival, remission). A recent review that catalogs “under-recognized adjunctive therapies” cautions that while such approaches are potentially useful, rigorous data remains scant and individualized tailoring is essential (27). Additionally, the possibility of placebo effects and patient expectation influencing subjective outcomes, particularly quality-of-life measures, cannot be excluded given the intensive and supportive nature of the interventions. Another important limitation is the absence of molecular or genomic biomarker analysis, which restricts mechanistic insights and limits the ability to correlate clinical responses with underlying biological changes.

Moreover, long-term follow-up is necessary to determine the durability of remission. Further, it should be noted that, these interventions should not replace conventional oncologic therapies; rather, they must be viewed as adjuncts aimed at improving the host’s metabolic, immunologic, and psychosocial resilience, potentially enhancing outcomes when combined with standard care. This aligns with the guiding principles of evidence-based integrative oncology. The patient expressed satisfaction with the outcomes achieved, noting significant improvements not only in his quality of life but also in the control of disease progression.

Nevertheless, this case contributes valuable insights to the limited body of literature on integrative management of bladder cancer. It advocates for the need to explore more inclusive oncological paradigms, where metabolic, psychological, and environmental factors are addressed alongside genetic and histological characteristics. Given the economic burden of bladder cancer treatment, especially in low-resource settings, cost-effective and evidence-informed integrative approaches may offer sustainable alternatives or adjuncts to conventional care.

Further, the improvements observed in biochemical markers, patient-reported outcomes, and imaging findings may reflect the combined effects of systemic therapy, supportive care, and individualized integrative interventions. However, as a single case report without controls, these findings are inherently descriptive and should be considered hypothesis-generating rather than evidence of efficacy.

An important clinical observation in this case was the absence of any clinically significant adverse interactions between the integrative interventions and the concurrent systemic anticancer therapies. The patient tolerated the combined multimodal approach well, without unexpected toxicities, treatment interruptions, or apparent compromise in the efficacy of systemic treatments. This finding is particularly relevant in the context of integrative oncology, where concerns regarding potential herb–drug or therapy–drug interactions often limit clinical adoption. While this single case suggests that a carefully monitored and individualized integrative approach may be feasible and well-tolerated alongside standard therapies, these observations should be interpreted with caution. Systematic studies are required to rigorously evaluate safety profiles, potential interactions, and generalizability across broader patient populations.

## Conclusion

This case underscores the potential of integrative oncology as a supportive approach along with systemic cancer therapies in advanced bladder cancer, leading to clinical remission and improved quality of life. By combining metabolic correction with targeted therapies, favorable outcomes were achieved without the toxicity commonly associated with standard treatments. While encouraging, these results warrant validation through larger, controlled studies.

## Data Availability

The original contributions presented in the study are included in the article/**Supplementary Material**. Further inquiries can be directed to the corresponding author.
