# Supplementary material for: Integrative oncology along with systemic cancer therapies in advanced bladder and prostate cancer: metabolic optimization guiding the shift from palliative care to clinical remission – a case report
Source: Front Oncol. 2026 May 18;16:1755406. doi: 10.3389/fonc.2026.1755406 (PMC13222838; doi:10.3389/fonc.2026.1755406)
Supplement: Supplementary file 1 [file DataSheet1.docx]

**A: Supplementary Table: The treatment protocol**

| **Domain** | **Intervention** | **Dosage / Modality Details** | **Frequency & Duration** | **Clinical Rationale** |
| --- | --- | --- | --- | --- |
| **Systemic Anticancer Therapy** | Afatinib (20 mg) | Oral, 1 tablet/day | Three times weekly for 6 months | EGFR inhibition; targeted anticancer effect |
|  | Axitinib (5 mg) | Oral, 1 tablet/day | Three times weekly for 6 months | VEGF inhibition; anti-angiogenic therapy |
|  | Abiraterone acetate (250 mg) | Oral, 2 tablet/day | Daily for 6 months | Androgen suppression; hormonal modulation |
|  | Relugolix (120 mg) | Oral, 1 tablet/day | Daily for 6 months | LHRH antagonist; hormonal suppression |
|  | Artesunate (IV) | 60–120 mg IV infusion | Once weekly (with IV vitamin C) | Proposed cytotoxic and anti-proliferative effects |
|  | High-dose Vitamin C (IV) | Total ≤90 g/week | 2–3 times per week | Pro-oxidant effect; adjunctive cytotoxic support |
| **Energy-Based / Biophysical Therapies** | Oncothermia (EHY-2030) | Modulated electrohyperthermia | Alternate days; 24 sessions (2 cycles with 2-week break) | Tumor-selective heating; apoptosis induction |
|  | PEMF Therapy (Pulsatron, Madras Institute of Magnetobiology, Chennai, India) | Pulsed electromagnetic field exposure | Daily, 30 min for 21 days | Cellular signaling modulation |
|  | Hydrosun Therapy (Hydrosun Medizintechnik, Müllheim, Germany) | Water-filtered infrared A irradiation | Daily, 20 minutes | Microcirculation enhancement |
|  | Hydrogen Inhalation (Athena Technology, Mumbai, India)   | Inhaled molecular hydrogen (1000 ml/min) | Daily, 30 minutes | Antioxidant and anti-inflammatory effects |
| **Detoxification & Metabolic Therapies** | Ethylenediaminetetraacetic acid (EDTA) Chelation | IV chelation therapy | Once every 15 days | Heavy metal detoxification (supportive rationale) |
|  | Ozone Therapy | Rectal/urethral insufflation, ozonated saline, Minor-autohemotherapy | Daily | Immunomodulation and oxidative preconditioning |
|  | Far Infrared Sauna / Ozone Steam Bath | Thermal detox modality | Once every 15 days | Detoxification and circulation support |
|  | Coffee Enema | 500 ml preparation (from 3 tbsp coffee/liter) | Once weekly | Proposed hepatic detoxification support |
| **Nutritional & Metabolic Support** | Diet Therapy | Millet-based, no refined sugar/wheat; fruits, vegetables, soups; 14:10 fasting | Daily | Metabolic regulation; insulin modulation |
|  | Liposomal Curcumin (250 mg per 5 ml) | 15 ml oral | Daily | Anti-inflammatory and anti-neoplastic potential |
|  | Coenzyme Q10 (Liposomal, 250 mg per 5 ml) | 5–15 ml oral | Daily | Mitochondrial support |
|  | Probiotic Supplementation | 15 billion CFU combination of Bifidobacterium and Lactobacillus genus (Livbiomes Probiotics, , Livbio Pharma, Kerala, India) | Twice, Daily | Gut microbiome modulation |
|  | Vitamin D | 60,000 IU oral | Weekly (monitored with PTH, Ca²⁺) | Immune and metabolic regulation |
| **Mind-Body & Supportive Therapies** | Counselling | Structured sessions | Daily | Psychological support |
|  | Yoga Therapy | Pranayama, relaxation, loosening exercises | Daily | Stress reduction; neuroendocrine modulation |
|  | Acupuncture | Standard (SP6, ST36, LV3, etc.) + symptom-based points | As per clinical schedule | Symptom relief; autonomic regulation |
| **Local / Physical Therapies** | Mud Therapy | Direct mud application over abdomen | 15-day cycles | Symptomatic and detox support |

**B: Supplementary Figure: Timeline of events**

**
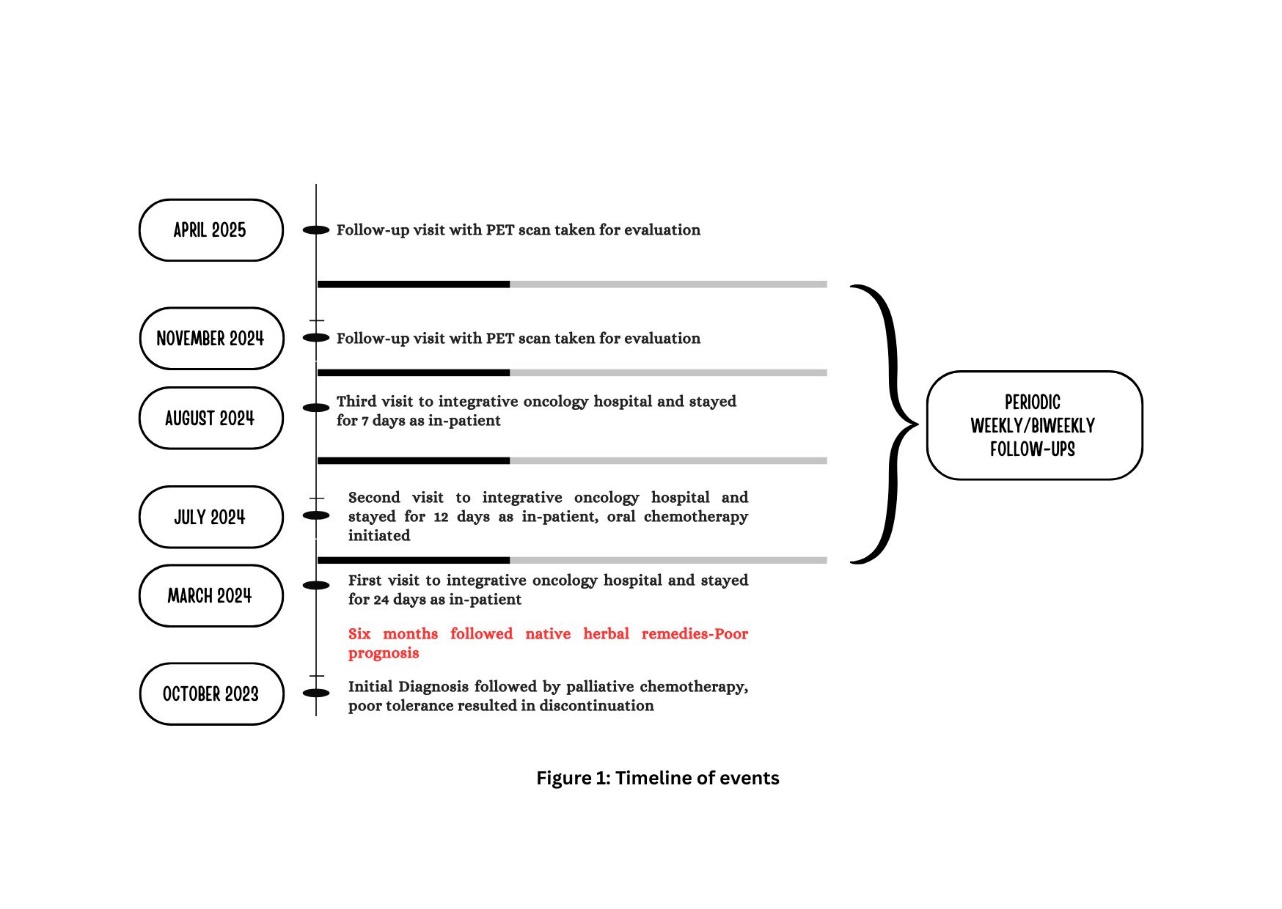
**
